# Screening synteny blocks in pairwise genome comparisons through integer programming

**DOI:** 10.1186/1471-2105-12-102

**Published:** 2011-04-18

**Authors:** Haibao Tang, Eric Lyons, Brent Pedersen, James C Schnable, Andrew H Paterson, Michael Freeling

**Affiliations:** 1 Department of Plant and Microbial Biology, University of California, Berkeley, CA, 94720, USA; 2 iPlant, Department of Plant Sciences, University of Arizona, Tucson, 85721, USA; 3 Plant Genome Mapping Laboratory, University of Georgia, Athens, GA, 30602, USA

## Abstract

**Background:**

It is difficult to accurately interpret chromosomal correspondences such as true orthology and paralogy due to significant divergence of genomes from a common ancestor. Analyses are particularly problematic among lineages that have repeatedly experienced whole genome duplication (WGD) events. To compare multiple "subgenomes" derived from genome duplications, we need to relax the traditional requirements of "one-to-one" syntenic matchings of genomic regions in order to reflect "one-to-many" or more generally "many-to-many" matchings. However this relaxation may result in the identification of synteny blocks that are derived from ancient shared WGDs that are not of interest. For many downstream analyses, we need to eliminate weak, low scoring alignments from pairwise genome comparisons. Our goal is to objectively select subset of synteny blocks whose total scores are maximized while respecting the duplication history of the genomes in comparison. We call this "quota-based" screening of synteny blocks in order to appropriately fill a quota of syntenic relationships within one genome or between two genomes having WGD events.

**Results:**

We have formulated the synteny block screening as an optimization problem known as "Binary Integer Programming" (BIP), which is solved using existing linear programming solvers. The computer program QUOTA-ALIGN performs this task by creating a clear objective function that maximizes the compatible set of synteny blocks under given constraints on overlaps and depths (corresponding to the duplication history in respective genomes). Such a procedure is useful for any pairwise synteny alignments, but is most useful in lineages affected by multiple WGDs, like plants or fish lineages. For example, there should be a 1:2 ploidy relationship between genome A and B if genome B had an independent WGD subsequent to the divergence of the two genomes. We show through simulations and real examples using plant genomes in the rosid superorder that the quota-based screening can eliminate ambiguous synteny blocks and focus on specific genomic evolutionary events, like the divergence of lineages (in cross-species comparisons) and the most recent WGD (in self comparisons).

**Conclusions:**

The QUOTA-ALIGN algorithm screens a set of synteny blocks to retain only those compatible with a user specified ploidy relationship between two genomes. These blocks, in turn, may be used for additional downstream analyses such as identifying true orthologous regions in interspecific comparisons. There are two major contributions of QUOTA-ALIGN: 1) reducing the block screening task to a BIP problem, which is novel; 2) providing an efficient software pipeline starting from all-against-all BLAST to the screened synteny blocks with dot plot visualizations. Python codes and full documentations are publicly available http://github.com/tanghaibao/quota-alignment. QUOTA-ALIGN program is also integrated as a major component in SynMap http://genomevolution.com/CoGe/SynMap.pl, offering easier access to thousands of genomes for non-programmers.

## Background

Many eukaryotic lineages have experienced whole genome duplication (WGD) events, including fungi [1], animals [2,3] and especially flowering plants, where WGDs are prevalent [4-6]. Over evolutionary time, evidence of WGDs is obscured by loss of duplicate genes, gene movement and genome rearrangements. Unequal evolutionary rates for different lineages and gene families further complicate this problem, making phylogenetic inferences from distributions of pairwise distances between paralogous genes difficult and occasionally leading to erroneous findings. Conservation of collinear gene order (or "synteny") is less subject than sequence conservation to difficulties with phylogenetic inference, and is the basis for the discovery and dating of ancient genomic events where whole genome sequence is available [6,7].

**Figure 1:**
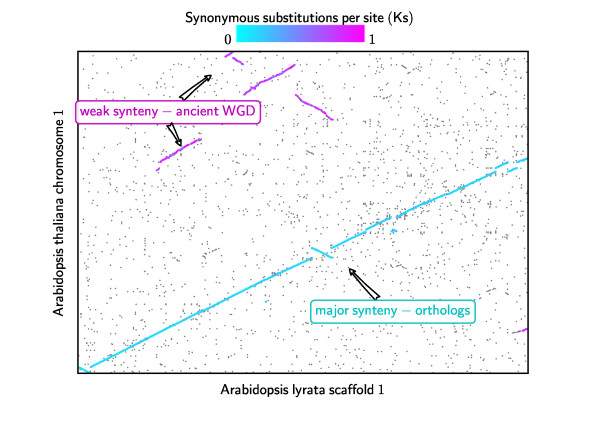
**Typical syntenic dot plot between genomes that have undergone shared ancient WGD events**. In this example, the synteny plot is between *A. lyrata *scaffold 1 (*x*-axis) and *A. thaliana *chromosome 1 (*y*-axis). Gray dots represent putative homologous gene pairs, and syntenic gene pairs are plotted with color based on their *Ks *values. Two significant patterns of synteny are evident. First, these genomes have syntenic regions identified by cyan color that are derived from the divergence of these two taxa, or orthologous blocks. Second, there are smaller magenta-colored synteny blocks that are derived from their shared WGD event and therefore older than orthologous blocks. We observe that these smaller, older blocks overlap with orthologous blocks along one or both of the genomes, which can be used as the basis for screening.

A typical pipeline for genome structure comparison starts with the enumeration of "synteny blocks" - regions of chromosomes between two or more input genomes that shared a common order of homologous genes and are therefore inferred to be derived from a common ancestor. Synteny blocks are often viewed as "diagonals" on a syntenic dot plot, where dots represent putative homologous gene pairs or marker pairs as inferred by sequence similarity (Figure 1).

WGDs can present a major challenge for accurately attributing synteny blocks to different evolutionary origins, especially when there are multiple sequential WGD events. In particular, some WGDs may be species-specific and others shared by multiple species (i.e. the polyploidy occurred before their lineages diverged). In order to automate the classification of different evolutionary origins for sets of syntenic blocks in lineages with a history of WGDs, it is essential to identify the relative ages of the WGDs and their placement relative to the divergence of the genomes being compared before performing in-depth analyses (see an example in Figure 1).

Current softwares to identify synteny blocks often uses chaining or clustering of putative homologous gene pairs [4,8-10]. It is common to then use custom schemes to score each block and apply a cutoff [8,11]. Existing methods do not differentiate the evolutionary origin and age of these synteny blocks. However, specification of identity and age is crucial for any downstream evolutionary analysis. These software packages for identifying syntenic blocks will identify some blocks that are derived from shared, ancient whole genome duplications as well as false syntenic regions created by repeats and local gene duplications, creating ambiguity in the identification of true syntenic orthologs.

Methods that find syntenic regions between vertebrate genomes often rely on "best-in-genome" (or "one-to-one" reciprocal best) criteria [12] in order to remove noise from the exhaustive enumeration of synteny blocks. This is not appropriate when studying plant or other genomes affected by multiple rounds of polyploidy. The orthologous blocks between two genomes under WGD scenarios can be one-to-many, or many-to-many depending on when the WGD(s) occurred in the evolutionary history of one or both lineages.

There have been *ad-hoc *rules in pairwise comparisons to extract synteny blocks under the influence of WGDs. For example, approaches that find "Doubly Conserved Syntenies" (DCS) were extensively used in yeast [1] and fish [3] genomes. The DCS method attempts to find 2 chromosomal regions that both match the same single region in the outgroup. However, it is only designed to work with the 2-to-1 case and does not deal with hexaploidy, double tetraploidy, or more general *n*-fold polyploidy. Additionally, the DCS method still relies on *ad-hoc *rules to classify the 2:1 pattern. Without an explicit optimization objective, the DCS method is not fully reproducible. DCS also requires the sequence of an unduplicated outgroup species, a resource not available in many cases.

To generalize the concept behind DCS, we observe that when aligning two genomes with known polyploidy events in one or both lineages, we often have expectations for the number of subgenomes, or "multiplication level", or "depths" [6,7,13,14]. Such *a priori *information can be used as a guide to screen synteny blocks. If we set up an upper bound ("quota") for the expected number of subgenomes, the synteny blocks will then "compete" for the specified depths and the identification of synteny blocks will then be selective, excluding weaker matches that are more ancient or simply artifacts. As more flowering plant lineages with complicated polyploidy histories are sequenced, tools to automate the identification of synteny blocks with known evolutionary origins will become valuable to a wider range of researchers.

The algorithm we present here, called QUOTA-ALIGN, is a method that screens synteny blocks based on the expected number of subgenomes, effectively eliminating more ancient (weaker) or spurious alignments. In its simplest usage, a quota of 1:1 between genomes of different species corresponds to orthologous blocks in the traditional sense, i.e. neither of the genomes have duplicated since their divergence (e.g. between two mammalian genomes) and will contain an approximately 1:1 syntenic mapping of genomic regions. In QUOTA-ALIGN, the quota constraint is generalized to *Q*_*X*_:*Q*_*Y *_in order to handle lineages with different duplication histories, a case found frequently in flowering plant lineages.

The quota-based screening of synteny blocks is a difficult problem because the goal is to simultaneously maximize synteny blocks' scores on both the *x*-axis and *y*-axis in a dot plot [15], where simple sorting along any one axis will not necessarily be optimal on the other axis. Even in the case of 1:1 quota, the problem is known to be NP-hard [15]. Herein, we show that it is possible to translate the problem into "Binary Integer Programming" (BIP), which is well-studied and has efficient software implementations. After converting to a BIP problem and solving it, QUOTA-ALIGN produces cleaner sets of synteny blocks and eliminates most ambiguous matches.

## Results

### Simulations

For the first test, we simulated genome evolution *in silico*, and tested whether QUOTA-ALIGN is capable of recovering true orthologous anchors between two genomes under the influence of genomic rearrangement events. We start with two genomes *A *and *B *with the same gene content *x*_*1 *_... *x*_*n *_where genes *x*_*i *_≠ *x*_*j *_for all 1 ≤ *i*, *j *≤ *n*, *i *≠ *j *- and simulate evolving genomes as signed permutations of gene symbols. Next, we simulated polyploidy events in both genomes such that genome *A *receives a duplication (×2), and genome *B *receives a triplication (×3). Effectively, each whole genome gets *k *copies (*k *= 2 or 3) that is concatenated and treated as one chromosome. Additionally, we simulated the following mutational events for a total of *N *steps: 1) macro-inversions - two breakpoints are randomly selected and the intervening chromosome segment is flipped; 2) gene losses - a randomly chosen gene symbol is deleted from the genome. At each step, we set the two mutation events to have different probabilities of occurring *P*_*inversion *_and *P*_*loss*_, or staying unchanged. True homologous gene pairs between genomes A and B are tracked throughout the simulation and are used as a "gold standard" to evaluate the pairs recovered by QUOTA-ALIGN. Formally, the recovery rate of true homologs can be calculated:

We set *N *= 20000 and vary *P*_*loss *_and *P*_*inversion*_, to investigate whether QUOTA-ALIGN is robust against different levels of genomic mutations. Since we know *a priori *that the quota ratio is 2:3, we used this information to screen the synteny blocks. With this simple simulation, we find that both gene losses and inversions affect the accuracy of QUOTA-ALIGN. While inversions have a slightly larger impact on the recovery rate of true homologs (Table 1), the recovery rate never drops below 80% for large probabilities of *P*_*inversion *_(0.02) and *P*_*loss *_(0.9).

**Table 1:** Impact of gene loss (*P*_*lo*__*s*__*s*_) and chromosomal inversions (*P*_*inversi*__*o*__*n*_) on the performance of QUOTA-ALIGN using the simulated genomes.

	***P***_***loss***_** = 0**	***P***_***loss***_** = 0.3**	***P***_***loss***_** = 0.6**	***P***_***loss***_** = 0.9**
*P*_*inversion *_= 0	100.0	100.0	100.0	100.0
*P*_*inversion *_= 0.005	99.6	99.5	99.3	98.6
*P*_*inversion *_= 0.01	97.9	97.6	97.2	94.3
*P*_*inversion *_= 0.015	95.5	94.2	90.9	89.6
*P*_*inversion *_= 0.02	91.3	89.4	86.6	80.3

For each case of varying *P*_*lo*__*s*__*s *_and *P*_*inversi*__*o*__*n*_,, we calculate the percentage of true homolog pairs recovered by QUOTA-ALIGN (100.0 means that every homolog gene pair between the two simulated genomes is identified by QUOTA-ALIGN).

### Estimating quota ratios

**Figure 2:**
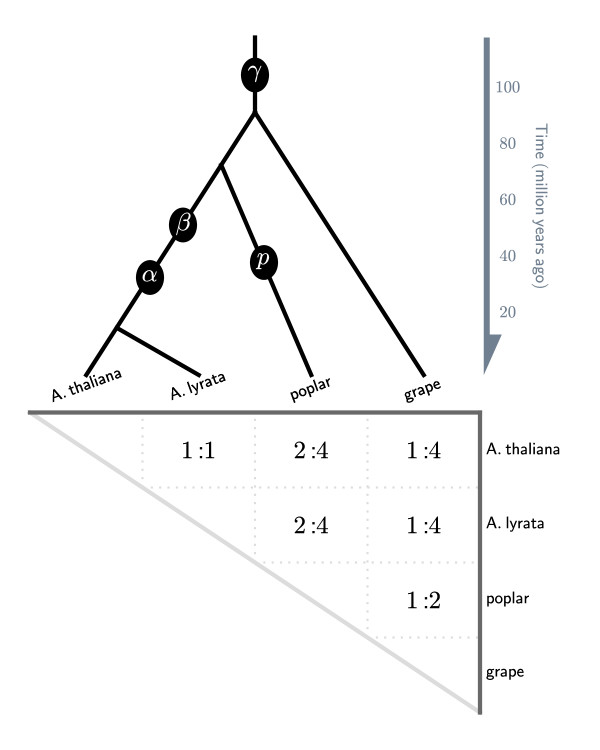
**Phylogeny for the rosid genomes used as test cases in this study, with known ancient WGDs marked on the branches**. Phylogeny of rosid genomes with circles denoting polyploidy events. α, β and ρ: tetraploidies; γ: hexaploidy. The quota displayed in the table are in the form of *Q*_*X*_*:Q*_*Y*_, where *Q*_*X *_is the expected multiplication level for the genome on the top of the table, and *Q*_*Y *_is the expected multiplication level for the genome on the right.

Selection of the quota ratio to use in comparisons of real genomes requires knowledge of the duplication histories of both species. For the identification of purely orthologous blocks between two genomes, *X *and *Y*, the first value of the quota ratio is the product of all polyploidy events unique to the *X *lineage since its divergence from the common ancestor of *X *and *Y*, and the second value is the product of all polyploidy events unique to the *Y *lineage. The quota ratio can be better understood with known polyploidy events mapped onto the species tree (Figure 2).

QUOTA-ALIGN can be used to identify paralogous syntenic blocks in addition to orthologous blocks. To identify paralogous blocks, the first and second values of the quota ratio are both multiplied by the product of one or more shared polyploidy events in the common ancestor of *X *and *Y*. To identify paralogous syntenic blocks within a single genome, both values of the quota ratio are the expected multiplication level minus 1 (to account for the self-self match). If the most recent polyploidy within a lineage is genome doubling, every genomic region will match one other region within the same genome (excluding the self-self match), therefore the expected quota is 1:1. If the most recent polyploid event is a genome triplication, every genomic region will match two other regions within the same genome, giving a quota ratio of 2:2.

### Applications to real data

We performed comparisons among four rosid genomes with a known phylogeny and polyploidy history (Figure 2). These genomes were selected to showcase the various genome comparisons that can benefit from quota-based screening. All four rosid genomes share a common genome triplication event called γ, and in some individual lineages there are subsequent genome doubling events [7]. Two exemplar cases are provided for screening orthologous and paralogous blocks, illustrating both "one-to-one" and "multiple-to-multiple" scenarios. Although examples are taken from plant genome comparisons, the algorithm in QUOTA-ALIGN is generic and is not limited to plant genomes. Finally, the plant genomes that are used in this study were downloaded from Phytozome http://www.phytozome.net, and all analysis results can be reproduced in the CoGe comparative genomics platform using its tool, SynMap (Table 2).

**Table 2:** Summary of synteny blocks before and after quota-based screening in the four examples used in this study.

Example		# of anchors	# of blocks	Percentage of regions exceed quota	Run time	CoGe link
*A. thaliana *vs. *A. lyrata *(1:1)	before	27381	388	76.3%	0.73s	http://bit.ly/aT6Uyx
	after	20826	69	0.1%		
						
*A. thaliana *vs. poplar (4:2)	before	16257	1084	18.4%	0.55s	http://bit.ly/9qXnMg
	after	14839	910	1.9%		
						
*A. thaliana *vs. *A. thaliana *(1:1)	before	6523	440	22.0%	0.25s	http://bit.ly/akpvUh
	after	5477	315	3.5%		
						
grape vs. grape (2:2)	before	4459	270	9.0%	0.24s	http://bit.ly/bCSHZG
	after	4297	259	2.7%		

For each case, summary of blocks *before *screening is in the first row, and blocks *after *screening is in the second row. The running time for QUOTA-ALIGN is based on a single-threaded 3.0 GHz Intel Xeon CPU.

#### Example 1: Orthologous blocks between A. thaliana and A. lyrata (quota 1:1)

**Figure 3:**
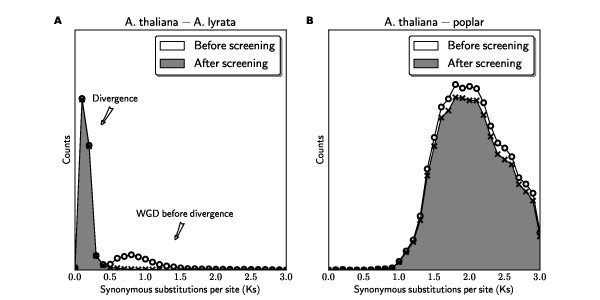
**Ks distributions for gene pairs before and after QUOTA-ALIGN**. Quota-based screening for our test cases: (A) *A. thaliana *- *A. lyrata *comparison; (B) *A. thaliana *- poplar comparison. In both cases, gene pairs that are derived from more ancient duplications were eliminated after QUOTA-ALIGN.

For finding orthologous blocks between *A. thaliana *and *A. lyrata *(available http://www.phytozome.net/alyrata), we first note that there are no genome duplications since their divergence (Figure 2), and therefore we need to enforce the quota ratio of 1:1 to identify orthologous syntenic blocks. The noise for this analysis is due to synteny blocks derived from shared WGDs prior to their divergence (Figure 2). Before quota screening, a total of 688 linear constraints are identified in this problem. After the 1:1 screening, most synteny blocks from the older WGDs are rejected as weaker matches (see an example for chromosome pair *A. thaliana *chromosome 1 vs. *A. lyrata *scaffold 1 in Figure 1) identifying 69 putatively orthologous syntenic blocks. As an independent validation, there were originally two major peaks in the *Ks *distribution and the older *Ks *peak (that correspond to the older WGD events) disappeared after the screening (Figure 3A).

#### Example 2: Orthologous blocks between arabidopsis (A. thaliana) and poplar (P. trichocarpa) (quota 4:2)

Comparison of *A. thaliana *and poplar illustrates a "multiple-to-multiple" case, i.e. where both genomes have experienced duplications since their divergence (Figure 2). We know *a priori *that *A. thaliana *had 2 sequential tetraploidies, while poplar had 1 tetraploidy since diverging from their most recent common ancestor; therefore the expected quota is 4 (2^2^) *A. thaliana *regions to 2 (2^1^) poplar regions. After 4:2 screening, relics of older WGD events (γ) in their shared lineage are removed, and only putative orthologous blocks are retained. Compared to Example 1, the higher quota ratio (4:2) in this example represents a much looser constraint. Therefore more blocks are kept, as also reflected in the smaller number of constraints - in this example there are a total of 1084 variables, but only 326 constraints.

#### Example 3: Arabidopsis duplicated blocks (quota 1:1)

Arabidopsis has undergone three WGD events and traces of these events are evident on the arabidopsis-arabidopsis dot plot [4] (Figure 2). The synteny blocks, if unscreened, will include both recent duplicates (α event) and older duplicates (β and γ event), which are difficult to differentiate. Bowers *et al*. (2003) manually curated a list of arabidopsis blocks involved in the most recent duplication (α event) (Figure 2), applying an implicit rule to always use the higher scoring synteny segments when two synteny segments are conflicting [4]. With QUOTA-ALIGN, we formulate this rule more explicitly by specifying our quota 1:1 when finding synteny blocks within the arabidopsis genome. The final problem contains 440 variables and 152 constraints. The final blocks we find are mostly consistent with Bowers' manually identified blocks and are in agreement with 94% of Bowers' data (3581 out of 3822 total pairs). However, QUOTA-ALIGN discovered an additional 1153 gene pairs that were missed in Bowers' data. We further note that some of our additional gene pairs are microRNA genes that were only recently added to the latest Arabidopsis annotation after Bowers' study. In addition, we found two small alpha syntenic blocks that were entirely missed in Bowers' curated set of duplicates.

#### Example 4: Grape triplicated blocks (quota 2:2)

The grapevine genome contains many triplet sets of syntenic regions, suggesting its genome is derived from a triplication (also known as the γ event) [4,5] (Figure 2). Therefore, for each grape region, there are two additional matching grape regions and so we enforce a quota of 2:2 in QUOTA-ALIGN. The γ event is the only known polyploidy event in the grape lineage, and has very few overlapping blocks. The problem contains 270 variables and only 12 constraints.

Results from the four examples are summarized in Table 2. Following the quota-based screening, the retained blocks conform more closely to expected ratios. Before the screening, there are many overlapping synteny blocks that exceed the expected ratio, ranging from 9% to 76% of the total blocks. After the screening, in all four examples there are fewer than 5% of the genomic regions that exceed the given quota. There are still minimal overlaps after the screening, because of the relaxation of "strict overlapping" with the *N*_*m *_parameter (see Methods).

## Discussion

### Choice of quota

Selecting an appropriate quota ratio represents the single most important parameter affecting the outcome of screening syntenic regions. A loose quota (ploidy level set too high) imposes fewer constraints on overlapping syntenic blocks and leads to incomplete screening. An overly stringent (too low) quota, on the other hand, might accidentally remove some relevant synteny blocks, resulting in over-screening. Currently, the user must specify the quota ratio used by QUOTA-ALIGN; therefore the user must know *a priori *the polyploidy history of the genomes to be compared.

In cases where the polyploidy history is unknown, one strategy is to use different quota ratios (e.g. starting with 1:1, then 1:2, 2:1, etc.) to screen the synteny blocks and then inspect the dot plot to see if major features are included. To aid the user in selecting the quota correctly, QUOTA-ALIGN reports statistics on coverage that can be used by the user to validate the choice of quota. For example, in the sorghum-maize comparison, where maize has a WGD following the divergence of their lineages, a quota ratio of 1:2 should be used. Using a ratio of 1:1 instead only aligns 63% of the maize genome, this low percentage indicates that the user is likely missing one duplicated subgenome. Another helpful tip is to color syntenic gene pairs based on their *Ks *values (Figure 1). Such visualization can quickly identify shared or independent WGD events, and identify syntenic regions of varied ages.

### Comparison of quota-based with divergence-based screening

As discussed above, most existing methods for screening syntenic blocks are based on *ad-hoc *rules. Some previous method uses frequency of synonymous substitutions (*Ks*) between syntenic gene pairs to differentiate the age of the blocks [16,17]. *Ks*-based methods are not always effective for distinguishing synteny blocks from different events because: 1) *Ks*-based screening is contingent on multiple genomic events that are separable on the *Ks *distribution; 2) even in cases where different events are separable, *Ks*-based screening still involves an arbitrary cut-off that best separates different events. This is further complicated by large variations of *Ks *distributions derived from a single event, temporally continuous gene duplications, and limited resolution of *Ks *rate estimates over long evolutionary distances.

In contrast, QUOTA-ALIGN seeks to maximize the coverage (reflected by the block range) as well as divergence (reflected by the block score). This assumption complements the approach of using *Ks *values as the proxies of age of the blocks. For example, the *Ks *distribution for *A. thaliana *- *A. lyrata *(Example 1) clearly shows multiple peaks and a cutoff value at *Ks *~0.5 can be applied in order to select only the orthologous gene pairs that are at lower *Ks *range (Figure 3A). In contrast, for the arabidopsis - poplar comparison (Example 2), the peak in the *Ks *distribution contains a mixture of orthologous and out-paralogous (derived from more ancient WGDs) gene pairs, with no conspicuous "saddle" point to be used as cut-off to select only orthologous blocks (Figure 3B). In summary, *Ks *is less effective at discriminating older evolutionary events when they occurred close together in divergence time, or a very long time ago, when synonymous substitutions saturate.

### Studying genome rearrangement events

With the GRIMM algorithm [18] and its web-based interface, we can analyze the possible rearrangement scenario between two genomes in an automated fashion. GRIMM calculates the number of inversions, translocations, chromosome fusions and fissions under a most parsimonious scenario. We further note that such rearrangement analysis is only possible with our screening of synteny blocks since the inclusion of blocks from more ancient genome duplications will confound the actual block order.

For example, with QUOTA-ALIGN, we found that there are 69 synteny blocks between *A. lyrata *and *A. thaliana *(Example 1 in Results). One most parsimonious solution to transform the order of these 69 blocks in *A. lyrata *to the order in *A. thaliana *involves 34 steps -4 chromosome fusions, 3 translocations and 27 inversions. Previous results based on the genetic map between *A. lyrata *and *A. thaliana *suggested fewer steps: 3 fusions, 2 translocations and 5 inversions [19]. Clearly the genome sequences offer higher resolutions than marker-based genetic maps, especially for finding small inversions. However, the higher number of rearrangements suggested by QUOTA-ALIGN might also be due to relative incompleteness of the current *A. lyrata *genome assembly. For example, the additional fusion event suggested by QUOTA-ALIGN appears to be caused by two large scaffolds that failed to join in the genome assembly.

One limitation of GRIMM is that it can only solve non-duplicated blocks. The reason for this is that the reconstruction of rearrangement history involving duplicated blocks is difficult algorithmic problem known as the "genome halving" for tetraploid genomes, or "genome aliquoting" for *n*-fold polyploid genomes [20]. These problems remain open and are only solved under strict assumptions. As such, the rearrangement analysis for ratios different than 1:1 is currently not supported in QUOTA-ALIGN.

### Scalability

Since the binary integer programming is NP-hard, the worst-case execution time increases exponentially with larger problem size. The size of the integer programming problem is determined by the number of binary variables (*n*) and constraints (*m*). However, due to the branch-and-bound and other heuristics employed by the solvers, the average running time is often dependent on the structure of particular data and not entirely predictable. We typically screen <1000 synteny blocks with the integer solver taking at most a few seconds to solve on a single-threaded 3.0 GHz Intel Xeon CPU. In all examples used in the Results, the screening step was able to finish within 1 second (Table 2).

QUOTA-ALIGN can also handle the Multiple Alignment Format (MAF) which is often the direct output from BLASTZ/LASTZ [21]. This allows us to perform synteny block selection on the nucleotide alignment blocks as well. However, it is not yet scalable for the solver to solve the problem instance except for small genomes or chromosome pairs, since BLASTZ output between two large genomes are typically on the order of ~100,000 blocks even after chaining (Kent, et al., 2003), which is prohibitively expensive for integer programming solvers on current computers.

We need to point out that the scalability issue is dependent on the integer solver that we used. We rely on open-source integer solvers (SCIP and GLPK) in our program, but faster commercial solvers (e.g. CPLEX or GUROBI) could be used when available with only minor modifications to the linear programming interface in QUOTA-ALIGN. Furthermore, when an exact method is too costly, approximate methods could be applied for dealing with large number of synteny blocks and constraints.

## Conclusions

Synteny identification and attribution to specific genomic events in an automated fashion remains a nontrivial task [22]. QUOTA-ALIGN solves the problem of selecting a subset of synteny blocks by using the expected quota known *a priori *from the inferred occurrences of past WGD events. This permits the user to identify subset of synteny blocks more relevant to a specific evolutionary event (e.g. species divergence or genome duplications). Quota-based screening is a good alternative to the *Ks*-based classification methods and is always superior when multiple genomic events cannot be distinguished by *Ks*.

## Methods

**Figure 4:**
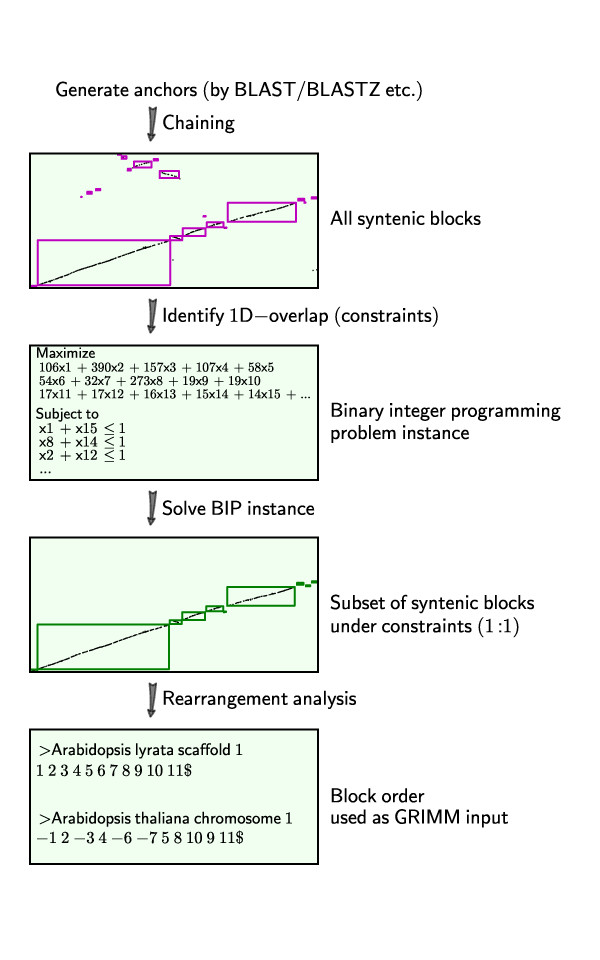
**QUOTA-ALIGN pipeline for analyzing synteny blocks**. One pair of chromosomes - *A. thaliana *chromosome 1 and *A. lyrata *scaffold 1 is used here to illustrate the multiple steps involved.

Here we describe the pipeline for identifying *Q*_*X*_:*Q*_*Y *_constrained quota depths. Briefly, we generate putative homologous anchors in two input genomes, group them into synteny blocks and screen the blocks based on the given constraints. We describe each step in further details, with an illustrative diagram for each individual step in Figure 4. We further note that our pipeline is modular in design so that at each step, the built-in algorithm can be replaced by other software with the appropriate format.

### Chaining synteny anchors to generate all syntenic blocks

First, the anchors (gene or marker pairs that show sequence similarities) can be generated by sequence alignment software like BLAST [23] or BLASTZ [21] based on all-against-all comparison between gene sequences of genome *X *and genome *Y*. Local gene duplicates and matches to multiple alternative splicing isoforms of the same gene are removed before chaining. Multiple models tend to place heavier weights on only a few genes and produce artifacts. The weight associated with each anchor reflects the similarity level for the match. We often use a scoring scheme for BLAST matches that transform the *E*-value to a score between 0 and 50, *S *= min (50, -log(*E*-value)), but other empirical scoring schemes are accepted. The sum of the weights of the anchors represents the weight of the synteny blocks: these weights are used in the screening of blocks.

**Figure 5:**
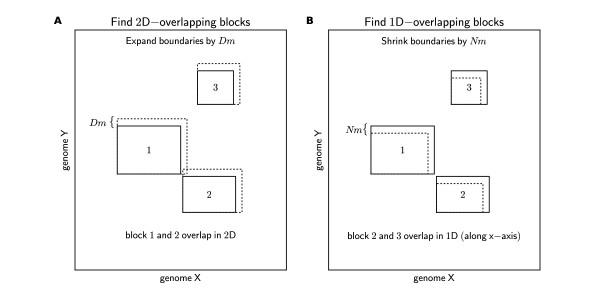
**Diagrams showing the 2D-overlap and 1D-overlap of synteny blocks**. *D*_*m *_and *N*_*m *_are the distance cutoffs that were used to correct the boundaries of the synteny blocks (dashed lines denote new boundaries). The correction of block boundaries allows more efficient implementation of overlap detection in both chaining and screening of synteny blocks.

QUOTA-ALIGN contains a general chaining algorithm to group the anchors into synteny blocks, similar to the idea used in GRIMM-synteny [18]. We make no distinction between the input data that are point types (*x*-position, *y*-position), or interval types (*x*-start, *x*-stop, *y*-start, *y*-stop), since we consider point anchors as degenerate interval anchors, only with the same start and stop. When two genomes under comparison are laid out on a 2D dot plot, the interval anchors represent rectangles on the synteny plot. The chaining algorithm then merges all "adjacent" rectangles up to a certain threshold distance *D*_*m*_. To simplify the implementation, we first expand the endpoints for all our blocks by *D*_*m *_(Figure 5A). This converts the problem from finding "adjacent" to "overlapping" blocks, for which there is an efficient sweep line algorithm described by Six and Wood [24].

### Identification of 1D-overlapping syntenic blocks to find conflicts

With genome duplications, synteny blocks that are distinct on the 2D synteny plot might still overlap in their projections onto the 1D axes along one or both genomes (Figure 1) [25]. If all duplicated regions are retained, the depths of their projection onto 1D axes (*x*-axis and *y*-axis) should reflect the duplication history. Identifying these 1D-overlapping blocks is thus essential for the subsequent screening of the synteny blocks. If at a certain position, the number of overlapping blocks exceeds the expected number of duplicated regions, then we probably should exclude some weaker blocks derived from more ancient events.

Before we describe the 1D-overlapping detection algorithm, we need to consider the scenario when there are synteny blocks that are only slightly overlapping but are blocks we do not want to exclude. This overlap is due to the over-extension in the previous chaining step. Therefore, we relax the criteria and specify a distance cutoff (*N*_*m*_) below which blocks are not considered overlapping.

Using a similar concept as employed in the chaining step, we again correct the boundaries of the synteny blocks. But this time instead of expanding, we shrink all our block boundaries by *N*_*m *_(Figure 5B). This boundary correction procedure simplifies the identification of the 1D-overlap using the aforementioned Six and Wood sweep line algorithm [24]. The set of overlapping blocks are then converted into constraints in the integer programming instance discussed in the following paragraph.

### Binary integer programming formulation

Linear programming is a method for finding the values that optimize a given model where all the constraints are represented as linear equations. The binary integer programming (BIP) problem is a special case where all the variables are required to be either 0 or 1. This is a well-known NP-hard problem [26].

Our problem formulation for QUOTA-ALIGN is the following: given the expected depth (quota) along the genomes on both *x*- and *y*-axis, select a subset of the synteny blocks with maximized total sum of scores of the selected blocks. We introduce a decision variable *x*_*i *_to determine whether or not we select each block *S*_*i*_. Let *w*_*i *_be the weight (or score) for synteny block *S*_*i *_for a total of n blocks, where *w*_*i *_is the sum of the weights in all the anchor points within *S*_*i*_. The binary integer formulation for our problem can thus be represented in a standard form:

• Objective function has the form maximize ∑ *w*_*i *_*x*_*i*_, for *i *= 1,2,... *n*

• The *m *constraints are inequalities of the form ∑ *x*_*j *_≤ *Q*_*X *_and ∑ *x*_*k *_≤ *Q*_*Y *_where the set of blocks they represent are overlapping on the *x*-axis and *y*-axis, respectively; *Q*_*X *_and *Q*_*Y *_represent expected numbers of subgenomes in genome *X *and genome *Y*.

• All of *x*_*i *_where *i *= 1,2,... *n *are binary variables to represent decisions (0 to discard the block, 1 to retain the block)

Note that in the simplest case when the quota is 1:1, the problem is the same as choosing a maximum weight independent set [15] in a conflict graph where edges represent 1D conflicts identified in the previous step.

### Solving the integer programming problem

After converting constraints of the synteny blocks to BIP problem, we use two free solvers - SCIP http://scip.zib.de/ and GLPK http://www.gnu.org/software/glpk/ to solve the instance. Our default solver SCIP was chosen for its superior performance http://plato.asu.edu/ftp/milpf.html. Both SCIP and GLPK generate the same solution in all test cases. Based on the output from the BIP solver, we keep all synteny blocks whose indicator variables are determined as 1. These are the "screened" blocks that maximize the total weights of the blocks while respecting the required quota. After screening with quota *Q*_*X*_:*Q*_*Y*_, each region in genome *X *will have at most *Q*_*Y *_matching regions in genome *Y*, while each region in genome *Y *will have up to *Q*_*X *_matching regions in genome *X*, thereby fulfilling the constraints.

### Chromosome segmentations for studying rearrangements

In the simplest case of quota 1:1, QUOTA-ALIGN produces synteny blocks that do not overlap in 1D projections, which is essential for studying rearrangement history [25]. We can then process the synteny blocks and represent the order of the blocks as integer sequences (where the integers represent identifiers of distinct blocks). Signs for the individual blocks were automatically determined by inspecting the start and stop anchor of the synteny block, to reflect whether the two matching regions are in the same or the opposite orientation [18]. The signed integer sequences can then be exported to the GRIMM web server [27] to search for one most parsimonious rearrangement scenario (Figure 4).

### Integration with SynMap application

We have included pre- and post-processing scripts in the QUOTA-ALIGN software package, as well as scripts to visualize the final blocks that have passed the quota-based screening. However, most users still need to download and process each individual genome and gene annotations. CoGe's database provides an integrated solution for data management, contains updated genome sequences and gene annotations, and provides a suite of web-based tools for comparative genomics [28]. Therefore we integrated QUOTA-ALIGN within CoGe's SynMap application to allow easier non-programmatic access to thousands of prokaryotic and eukaryotic genomes.

**Figure 6:**
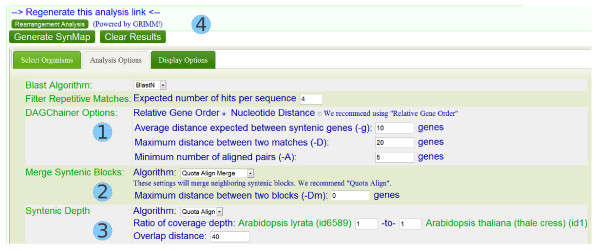
**Screenshot of analysis options on SynMap**. The whole synteny pipeline contains a few components, streamlined by QUOTA-ALIGN: 1) chaining anchors; 2) 2D overlapping block merging; 3) screening of synteny blocks. In the example shown, we are only interested in orthologous blocks between *A. thaliana *and *A. lyrata*, therefore a quota of 1:1 is used here. "Overlap distance parameter" is also known as *Nm *in the Methods; 4) encoding of the order for the screened blocks is automatically sent to the GRIMM server [27] for rearrangement analysis.

SynMap http://genomevolution.org/CoGe/SynMap.pl contains a set of tools within CoGe to compare genomes from many organisms, including identification of syntenic regions [29]. The QUOTA-ALIGN procedure may be invoked in the "Analysis Option" in the SynMap web interface (Figure 6). Users may select "Merge syntenic blocks" that chains syntenic blocks and "Syntenic depth" to specify the expected depths for screening of synteny blocks. The chaining and screening procedures are handled by QUOTA-ALIGN internally. Finally, rearrangement analysis through the GRIMM server is provided in the SynMap web interface (Figure 6).

## Authors' contributions

HT conceived the study, designed the algorithm and wrote the software. EL, BP, JS tested the software and performed the analysis. HT, AHP, MF drafted the manuscript. All authors read and approved the final manuscript.

## References

[B1] Kellis M Birren BW Lander ES Proof and evolutionary analysis of ancient genome duplication in the yeast Saccharomyces cerevisiae Nature 2004 428 6983 617 624 10.1038/nature02424 15004568

[B2] Aury JM Jaillon O Duret L Noel B Jubin C Porcel BM Segurens B Daubin V Anthouard V Aiach N Global trends of whole-genome duplications revealed by the ciliate Paramecium tetraurelia Nature 2006 444 7116 171 178 10.1038/nature05230 17086204

[B3] Jaillon O Aury JM Brunet F Petit JL Stange-Thomann N Mauceli E Bouneau L Fischer C Ozouf-Costaz C Bernot A Genome duplication in the teleost fish Tetraodon nigroviridis reveals the early vertebrate proto-karyotype Nature 2004 431 7011 946 957 10.1038/nature03025 15496914

[B4] Bowers JE Chapman BA Rong J Paterson AH Unravelling angiosperm genome evolution by phylogenetic analysis of chromosomal duplication events Nature 2003 422 6930 433 438 10.1038/nature01521 12660784

[B5] Jaillon O Aury JM Noel B Policriti A Clepet C Casagrande A Choisne N Aubourg S Vitulo N Jubin C The grapevine genome sequence suggests ancestral hexaploidization in major angiosperm phyla Nature 2007 449 7161 463 467 10.1038/nature06148 17721507

[B6] Van de Peer Y Fawcett JA Proost S Sterck L Vandepoele K The flowering world: a tale of duplications Trends Plant Sci 2009 14 12 680 688 10.1016/j.tplants.2009.09.001 19818673

[B7] Tang H Bowers JE Wang X Ming R Alam M Paterson AH Synteny and collinearity in plant genomes Science 2008 320 5875 486 488 10.1126/science.1153917 18436778

[B8] Haas BJ Delcher AL Wortman JR Salzberg SL DAGchainer: a tool for mining segmental genome duplications and synteny Bioinformatics 2004 20 18 3643 3646 10.1093/bioinformatics/bth397 15247098

[B9] Simillion C Janssens K Sterck L Van de Peer Y i-ADHoRe 2.0: an improved tool to detect degenerated genomic homology using genomic profiles Bioinformatics 2008 24 1 127 128 10.1093/bioinformatics/btm449 17947255

[B10] Soderlund C Nelson W Shoemaker A Paterson A SyMAP: A system for discovering and viewing syntenic regions of FPC maps Genome Res 2006 16 9 1159 1168 10.1101/gr.5396706 16951135 PMC1557773

[B11] Wang X Shi X Li Z Zhu Q Kong L Tang W Ge S Luo J Statistical inference of chromosomal homology based on gene colinearity and applications to Arabidopsis and rice BMC Bioinformatics 2006 7 447 10.1186/1471-2105-7-447 17038171 PMC1626491

[B12] Miller W Rosenbloom K Hardison RC Hou M Taylor J Raney B Burhans R King DC Baertsch R Blankenberg D 28-way vertebrate alignment and conservation track in the UCSC Genome Browser Genome Res 2007 17 12 1797 1808 10.1101/gr.6761107 17984227 PMC2099589

[B13] Kent WJ Baertsch R Hinrichs A Miller W Haussler D Evolution's cauldron: duplication, deletion, and rearrangement in the mouse and human genomes Proc Natl Acad Sci USA 2003 100 20 11484 11489 10.1073/pnas.1932072100 14500911 PMC208784

[B14] Tang H Wang X Bowers JE Ming R Alam M Paterson AH Unraveling ancient hexaploidy through multiply-aligned angiosperm gene maps Genome Res 2008 18 12 1944 1954 10.1101/gr.080978.108 18832442 PMC2593578

[B15] Bafna V Narayanan B Ravi R Nonoverlapping Local Alignments (Weighted Independent Sets of Axis Parallel Rectangles) Discrete Applied Mathematics 1996 41 41 53 10.1016/S0166-218X(96)00063-7

[B16] Cui L Wall PK Leebens-Mack JH Lindsay BG Soltis DE Doyle JJ Soltis PS Carlson JE Arumuganathan K Barakat A Widespread genome duplications throughout the history of flowering plants Genome Res 2006 16 6 738 749 10.1101/gr.4825606 16702410 PMC1479859

[B17] Simillion C Vandepoele K Van Montagu MC Zabeau M Van de Peer Y The hidden duplication past of Arabidopsis thaliana Proc Natl Acad Sci USA 2002 99 21 13627 13632 10.1073/pnas.212522399 12374856 PMC129725

[B18] Pevzner P Tesler G Genome rearrangements in mammalian evolution: lessons from human and mouse genomes Genome Res 2003 13 1 37 45 10.1101/gr.757503 12529304 PMC430962

[B19] Yogeeswaran K Frary A York TL Amenta A Lesser AH Nasrallah JB Tanksley SD Nasrallah ME Comparative genome analyses of Arabidopsis spp.: inferring chromosomal rearrangement events in the evolutionary history of A. thaliana Genome Res 2005 15 4 505 515 10.1101/gr.3436305 15805492 PMC1074365

[B20] Warren R Sankoff D Genome aliquoting with double cut and join BMC Bioinformatics 2009 10 Suppl 1 S2 10.1186/1471-2105-10-S1-S2 19208119 PMC2648758

[B21] Schwartz S Kent WJ Smit A Zhang Z Baertsch R Hardison RC Haussler D Miller W Human-mouse alignments with BLASTZ Genome Res 2003 13 1 103 107 10.1101/gr.809403 12529312 PMC430961

[B22] Catchen JM Conery JS Postlethwait JH Automated identification of conserved synteny after whole-genome duplication Genome Res 2009 19 8 1497 1505 10.1101/gr.090480.108 19465509 PMC2720179

[B23] Altschul SF Gish W Miller W Myers EW Lipman DJ Basic local alignment search tool J Mol Biol 1990 215 3 403 410 2231712 10.1016/S0022-2836(05)80360-2

[B24] Six HW Wood D The rectangle intersection problem revisited BIT Numerical Mathematics 1980 20 4 426 433 10.1007/BF01933636

[B25] Peng Q Alekseyev M Tesler G Pevzner P Decoding Synteny Blocks and Large-Scale Duplications in Mammalian and Plant Genomes Algorithms in Bioinformatics 2009 220 232 full_text

[B26] Karp RM Reducibility among combinatorial problems 1972 New York: Plenum

[B27] Tesler G GRIMM: genome rearrangements web server Bioinformatics 2002 18 3 492 493 10.1093/bioinformatics/18.3.492 11934753

[B28] Lyons E Freeling M How to usefully compare homologous plant genes and chromosomes as DNA sequences Plant J 2008 53 4 661 673 10.1111/j.1365-313X.2007.03326.x 18269575

[B29] Lyons E Pedersen B Kane J Freeling M The Value of Nonmodel Genomes and an Example Using SynMap Within CoGe to Dissect the Hexaploidy that Predates the Rosids Tropical Plant Biology 2008 1 3 181 190 10.1007/s12042-008-9017-y

